# Integrated Phenotypic and Transcriptomic Analyses of Osteoporosis in Type 2 Diabetic Mice

**DOI:** 10.7150/ijms.109537

**Published:** 2025-03-10

**Authors:** Shu-Juan Xing, Ying-Feng Gao, Lu Liu, Bing-Dong Sui, Ning-Ning Da, Jin-Yu Liu, Hao Wang, Yuan Yuan, Yuan Qin, Pei-Sheng Liu, Si-Qi Ying, Kai Zhang, Jie-Xi Liu, Ji Chen, Yi-Han Liu, Xin Xie, Yan Jin, Sha Zhang, Chen-Xi Zheng

**Affiliations:** 1 College of Life Science, Northwest University, Xi'an, Shaanxi 710069, China.; 2 State Key Laboratory of Oral & Maxillofacial Reconstruction and Regeneration, National Clinical Research Center for Oral Diseases, Shaanxi International Joint Research Center for Oral Diseases, Center for Tissue Engineering, School of Stomatology, The Fourth Military Medical University, Xi'an, Shaanxi 710032, China.; 3 Xi'an Key Laboratory of Stem Cell and Regenerative Medicine, Institute of Medical Research, Northwestern Polytechnical University, Xi'an, Shaanxi 710072, China.; 4 Department of Orthodontics, School of Stomatology, The Fourth Military Medical University, Xi'an, Shaanxi 710032, China.; 5 Department of Oral Implantology, School of Stomatology, The Fourth Military Medical University, Xi'an, Shaanxi 710032, China.; 6 Department of Stomatology, the First Medical Center, Chinese PLA General Hospital, Beijing, Beijing 100039, China.; 7 Key Laboratory of Resource Biology and Biotechnology in Western China, Ministry of Education, School of Medicine, Northwest University, Xi'an, Shaanxi 710069, China.; 8 College of Basic Medicine, Shaanxi Key Laboratory of Research on TCM Physical Constitution and Diseases Prevention and Treatment, Shaanxi University of Chinese Medicine, Xianyang, Shaanxi 712046, China.; 9 Department of Traditional Chinese Medicine, The First Affiliated Hospital of Fourth Military Medical University, Xi'an, Shaanxi 710032, China.

**Keywords:** Type 2 diabetes, bone, osteoporosis, transcriptome, mesenchymal stem cells

## Abstract

**Background:** Type 2 diabetes (T2D) is a global metabolic condition associated with complications of multiple organs, including the bone. However, the exact impact of T2D on bone along the disease progression, particularly in the early phase, remains largely unknown.

**Methods:** Four-week and sixteen-week high-fat diet (HFD) feeding-induced T2D mouse models were established, and the glucose metabolic status was examined. Bone mass was evaluated by micro-computed tomography (micro-CT), and immunofluorescent (IF) staining was performed for bone histomorphometry with enzyme-linked immunosorbent assay (ELISA) determining serum markers. RNA sequencing analysis was performed to examine the transcriptome of bone, and single-cell RNA-sequencing (scRNA-seq) analysis was further applied. Bone marrow mesenchymal stem cells (BMMSCs) were isolated and analyzed for functional behaviors.

**Results:** The occurrence of glucose metabolic disorders was confirmed at both four weeks and sixteen weeks of HFD feeding, showing increased blood glucose levels with impaired glucose tolerance and insulin sensitivity. Notably, early T2D osteoporosis symptoms were detected at four weeks, especially in the trabecular bone, demonstrating reduced bone mass and mineral density. Histological analysis confirmed that bone remodeling and immune-related inflammation were also altered in T2D mice, remarkably at the early phase, mainly reflected by suppressed bone formation, stimulated bone resorption, increased macrophages, and elevated tumor necrosis factor-alpha (TNF-α) levels. Transcriptomic sequencing further demonstrated significant yet distinct changes in the gene expression profile of bone during T2D progression, which confirmed the histological findings. Notably, overlapping genes with altered expression at four weeks and sixteen weeks of T2D compared to the respective control were identified, and bone marrow scRNA-seq analysis indicated many of them were expressed in BMMSCs, suggesting BMMSCs critically involved in T2D osteoporosis. Dysregulated molecular profiles and functional abnormalities of BMMSCs in T2D mice were validated by *ex vivo* assays, showing early and persistent occurrence of impaired colony-forming and proliferative capacities with biased differentiation potential.

**Conclusions:** These findings elucidate the bone lesion phenotype in T2D, particularly at the early phase, uncover changes in gene expression profiles of bone during T2D progression, and clarify the functional alterations in bone stem cells, providing a basis for subsequent research and the development of treatment strategies.

## Introduction

Type 2 diabetes (T2D) is a chronic metabolic disease worldwide characterized by high blood sugar (hyperglycemia) and high blood fat (hyperlipemia), which accounts for approximately 90-95% of all diagnosed cases of diabetes 1-3. The global prevalence of T2D has been rising steadily, posing significant health challenges and economic burdens 4, 5. T2D has been reported to lead to various complications affecting multiple organ systems 1, 6, 7. One significant complication that often arises in individuals with T2D is related to bone health 8, 9. Studies have shown that although bone mineral density (BMD) is close to normal or increased, T2D patients have an increased risk of fracture, suggesting that they have a recessive defect in bone quality 10-13. Animal studies have further revealed that T2D leads to impairment of skeletal quality and strength, but the timing of the occurrence of abnormal phenotypes of bone was slightly different among studies, possibly related to the inconsistency of the animal strains and modeling time selected for experimental models 14-19. Studies have particularly suggested that for the typical high-fat diet (HFD)-induced T2D mouse model, glucose and lipid metabolism abnormalities develop early after HFD feeding, and the bone complication occurs after around 8-16 weeks of feeding 20-22. However, there is still no research deciphering the potential bone alterations at the onset of T2D when the metabolic syndrome early develops.

The emergence of high-throughput sequencing technology has provided approaches to study overall molecular changes at the histological level, which enables the scientific community to gain a deeper understanding of bone physiology and pathology 23, 24. The cellular composition of the bone marrow microenvironment has been revealed by the single-cell RNA sequencing (scRNA-seq) technique 25-27, which in combination with histological sequencing technology will promote the exploration of underlying molecular mechanisms of bone health and disease. The homeostasis of bone relies on the balance between bone formation and bone resorption regulated by immune reactions 15, 28, 29. In specific, as an important stem cell population, bone marrow mesenchymal stem cells (BMMSCs) have been recognized as key players in bone homeostasis due to their ability to differentiate into osteoblasts, which are responsible for bone formation, and regulate osteoclast differentiation and activity, which are involved in bone resorption 30-32. It has been reported that the reduced bone quality and strength in T2D is accompanied by reduced bone turnover and that BMMSCs derived from diabetic individuals show impaired functions 17, 33. Revealing the phenotype of BMMSCs in the early phase of T2D, as well as the transcriptome profile, is crucial to further recognize the association between T2D and bone health and will help develop targeted interventions.

Here, based on previous studies 20-22, we established T2D mice models induced by different durations (4 weeks and 16 weeks) of HFD feeding. We confirmed that glucose metabolic disorders were aggravated in HFD mice at 16 weeks than at 4 weeks of feeding, indicating the T2D disease progression. Notably, the mice exhibited symptoms of osteoporosis in the early phase of T2D, primarily manifested in the trabecular bone. We discovered that in T2D, the osteogenic activity decreased, the osteoclastic activity increased, accompanied by an elevated level of immune-related inflammation, and these changes were more pronounced in the early phase. Transcriptomic sequencing further revealed that T2D caused significant alterations in the gene expression profile of bone, with distinct characteristics observed at different time points. We further combined scRNA-seq data to analyze at the single-cell expression level the genes that commonly changed in the bone transcriptomes of different time points of T2D, and found that many were expressed in BMMSCs. Moreover, BMMSCs isolated from the T2D mice and subjected to *in vitro* culture and testing showed that dysregulated molecular profiles and functional abnormalities of BMMSCs appeared early in T2D. Our study thus elucidated the bone lesion phenotype in T2D, particularly at the early phase, uncovered changes in gene expression profiles of bone during T2D progression, and clarified the functional alterations in bone cells, providing a basis for subsequent research and the development of treatment strategies.

## Results

### Glucose metabolic disorders in HFD-induced T2D mice at different time points

**Figure 1:**
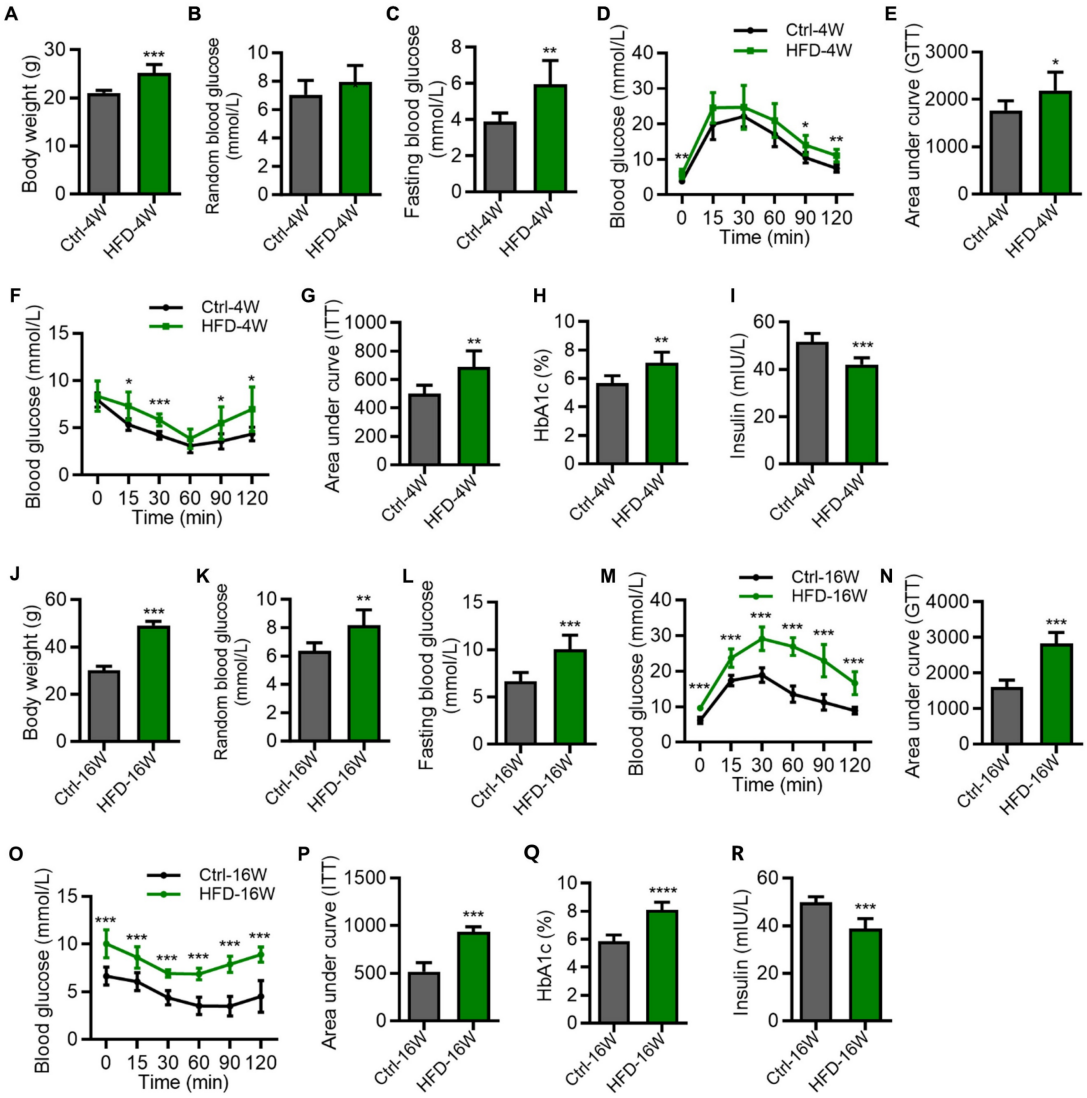
**Hyperglycemia in different time points of high-fat diet (HFD)-induced type 2 diabetes (T2D) mice.** (A-C) Body weight (A), random blood glucose (B) and fasting blood glucose (C) of HFD-feeding for 4 weeks (HFD-4W) and control (Ctrl-4W) mice. *N* = 6 per group. (D and E) Blood glucose levels during glucose tolerance tests (GTT) and quantification of area under the curve (AUC) of HFD-4W and Ctrl-4W mice.* N* = 6 per group. (F and G) Blood glucose levels during insulin tolerance tests (ITT) and quantification of AUC of HFD-4W and Ctrl-4W mice. *N* = 6 per group. (H and I) The serum percentages of HbA1c (H) and insulin levels (I) of HFD-feeding for HFD-4W and Ctrl-4W mice. *N* = 6 per group. (J-L) Body weight (J), random blood glucose (K) and fasting blood glucose (L) of HFD-feeding for 16 weeks (HFD-16W) and control (Ctrl-16W) mice.* N* = 6 per group. (M and N) Blood glucose levels during GTT and quantification of AUC of HFD-16W and Ctrl-16W mice. *N* = 6 per group. (O and P) Blood glucose levels during ITT and quantification of AUC of HFD-16W and Ctrl-16W mice. *N* = 6 per group. (Q and R) The serum percentages of HbA1c (Q) and insulin levels (R) of HFD-16W and Ctrl-16W mice. *N* = 6 per group. Mean ± standard deviation (SD). *, *P* < 0.05; **, *P* < 0.01; ***, *P* < 0.001. Student's two-tailed unpaired *t* tests.

We first established the T2D mice model by HFD feeding for 4 weeks and 16 weeks, and evaluated the related metabolic indexes. Emerging studies have suggested that for the HFD model, the metabolic syndrome develops as early as 4-week feeding, while the bone complication occurs after around 12-16 weeks of feeding 20-22. The results showed that compared with the control group, the mice fed with HFD for 4 weeks had a significant increase in body weight (Figure 1A). Compared with the control group, the random blood glucose of the HFD-4W group showed an upward trend, but the difference was not statistically significant (Figure 1B). The fasting blood glucose level was elevated (Figure 1C), and the symptoms of impaired glucose tolerance (Figure 1D and E) and insulin sensitivity (Figure 1F and G) were observed, along with an increased glycated hemoglobin (HbA1c) percentage and a decreased serum insulin level (Figure 1H and I).

As for the mice fed with HFD for 16 weeks, they had an extremely significant increase in body weight (Figure 1J), random blood glucose (Figure 1K), and fasting blood glucose level (Figure 1L), compared with the control group. Moreover, these mice exhibited significant reduction of glucose tolerance (Figure 1M and N) as well as insulin resistance (Figure 1O and P), accompanied by abnormal HbA1c and insulin levels (Figure 1Q and R). We note that the impairment of glucose and insulin tolerances of HFD mice was more prominent at 16 weeks than at 4 weeks (Figure 1D-G and M-P), indicating the T2D disease progression. Together, these results identified glucose metabolic disorders in HFD-induced T2D mice at different time points.

### Osteoporosis occurs early in the pathogenesis of T2D mice

**Figure 2:**
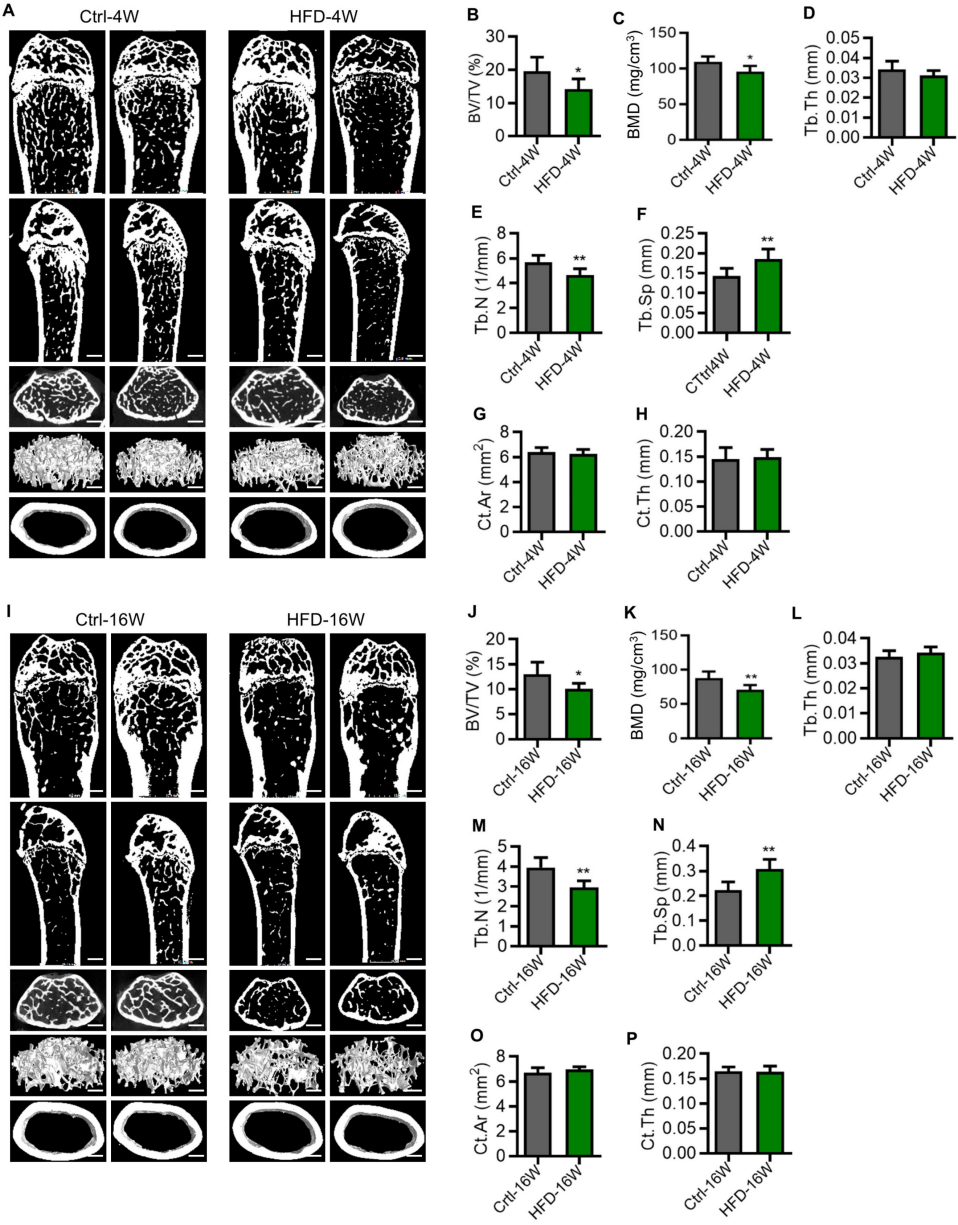
** Osteoporosis occurs early in the pathogenesis of type 2 diabetes (T2D) mice.** (A) Representative micro-CT images of the femora of HFD-feeding for 4 weeks (HFD-4W) and control (Ctrl-4W) mice. Scale bars, 500 µm. (B-F) Trabecular bone parameters of HFD-4W and Ctrl-4W mice were analyzed by micro-CT. BV/TV, bone volume over tissue volume (B); BMD, bone mineral density (C); Tb.Th, trabecular thickness (D); Tb.N, trabecular number (E); Tb.Sp, trabecular separation (F). *N* = 6 per group. (G and H) Parameters of cortical bone mass analyzed by micro-CT. Ct.Ar, surface area of cortical bone (G); Ct.Th, thickness of trabecular bone (H). *N* = 6 per group. (I) Representative micro-CT images of the femurs of HFD-feeding for 16 weeks (HFD-16W) and control (Ctrl-16W) mice. Scale bars, 500 µm. (J-N) Trabecular bone parameters of HFD-16W and Ctrl-16W mice were analyzed by micro-CT. *N* = 6 per group. (O and P) Parameters of cortical bone mass analyzed by micro-CT. *N* = 6 per group. Mean ± standard deviation (SD). *, *P*<0.05; **, *P*<0.01. Student's two-tailed unpaired *t* tests.

To investigate whether osteoporosis occurs in T2D, we next explored the bone phenotype at different time points of HFD feeding. T2D mice used in the experiments were selected based on their GTT. The femora of mice were scanned by micro-computed tomography (micro-CT). Compared to the control group, mice fed with HFD for 4 weeks showed significantly reduced bone mass (Figure 2A). This was mainly reflected in the reduction of trabecular bone volume over tissue volume (BV/TV) (Figure 2B), the reduction of BMD (Figure 2C), the reduction of trabecular number (Tb.N) (Figure 2E), and the increase of trabecular spacing (Tb.Sp) (Figure 2F), while the thickness of trabecular bone (Tb.Th) (Figure 2D), the surface area of cortical bone (Ct.Ar) (Figure 2G), and the thickness of cortical bone (Ct.Th) (Figure 2H) did not change. Similarly, we found that the bone mass was significantly reduced in mice fed with HFD for 16 weeks compared with the control group (Figure 2I), which was also mainly manifested in the reduction of BV/TV (Figure 2J), the reduction of BMD (Figure 2K) and Tb.N (Figure 2M), and the increase of Tb.Sp (Figure 2N), while the Tb.Th (Figure 2L), Ct.Ar (Figure 2O) and Ct.Th (Figure 2P) showed no significant difference. These findings suggest that T2D leads to reduced bone mass and impairment of bone microstructure, which were observed in the early phase. Furthermore, the pathological changes mainly occurred in the cancellous bone, with little effect on the cortex.

### Alterations of bone remodeling and immune-related inflammation in HFD-induced T2D mice

**Figure 3:**
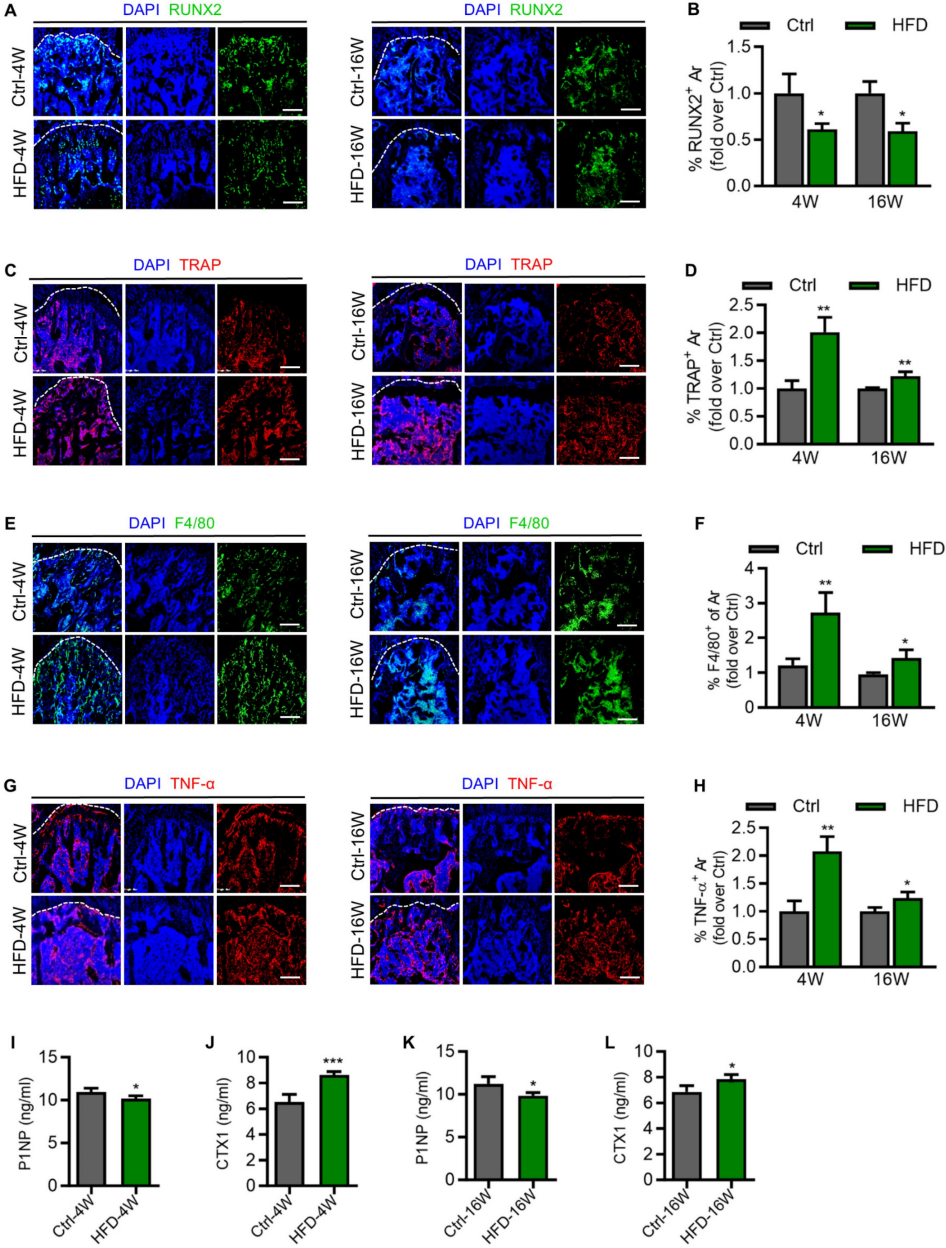
** Altered activity of bone formation, resorption and immune reaction in different time points of type 2 diabetes (T2D) mice.** (A) Representative immunofluorescent (IF) staining images of Runt-related transcription factor 2 (RUNX2) (green) in the femoral metaphysis of HFD-feeding for 4 weeks (HFD-4W) versus control (Ctrl-4W) mice and HFD-feeding for 16 weeks (HFD-16W) versus control (Ctrl-16W) mice, counterstained by DAPI (blue). Scale bars, 100 µm. (B) Corresponding quantification showing fold changes of the percentage of RUNX2^+^ area in HFD-4W compared to Ctrl-4W group and HFD-16W compared to Ctrl-16W group. *N* = 3 per group. (C) Representative IF staining images of Tartrate resistant acid phosphatase (TRAP) (red) in the femoral metaphysis of HFD-4W versus Ctrl-4W and HFD-16W versus Ctrl-16W mice, counterstained by DAPI (blue). Scale bars, 100 µm. (D) Corresponding quantification showing fold changes of the percentage of TRAP^+^ area in HFD-4W compared to Ctrl-4W group and HFD-16W compared to Ctrl-16W group. *N* = 3 per group. (E) Representative IF staining images of F4/80 (red) in the femoral metaphysis of HFD-4W versus Ctrl-4W and HFD-16W versus Ctrl-16W mice, counterstained by DAPI (blue). Scale bars, 100 µm. (F) Corresponding quantification showing fold changes of the percentage of F4/80^+^ area in HFD-4W compared to Ctrl-4W group and HFD-16W compared to Ctrl-16W group. *N* = 3 per group. (G) Representative IF staining images of tumor necrosis factor-α (TNF-α) (red) in the femoral metaphysis of HFD-4W versus Ctrl-4W and HFD-16W versus Ctrl-16W mice, counterstained by DAPI (blue). Scale bars, 100 µm. (H) Corresponding quantification showing fold changes of the percentage of TNF-α^+^ area in HFD-4W compared to Ctrl-4W group and HFD-16W compared to Ctrl-16W group. *N* = 3 per group. (I) The concentration of serum procollagen type 1 N-terminal propeptide (P1NP) in HFD-4W compared to Ctrl-4W group.* N* = 4 per group. (J) The concentration of serum C-terminal cross-linked telopeptide 1 (CTX1) in HFD-4W compared to Ctrl-4W group.* N* = 4 per group. (K) The concentration of serum P1NP in HFD-16W compared to Ctrl-16W group.* N* = 4 per group. (L) The concentration of serum CTX1 in HFD-16W compared to Ctrl-16W group.* N* = 4 per group. Mean ± standard deviation (SD). *, *P* <0.05; **, *P* <0.01; ***, *P* < 0.001. Student's two-tailed unpaired *t* tests.

As important indicators of bone remodeling, we next evaluated the activity of osteogenesis and osteoclastogenesis in T2D mice. Immunofluorescence (IF) staining results showed that expression of the osteogenic marker, Runt-related transcription factor 2 (RUNX2), was significantly reduced in the femora of HFD-4W and HFD-16W mice, compared to their respective control group (Figure 3A and B). In addition, expression of the osteoclast marker, Tartrate-resistant acid phosphatase (TRAP), was significantly increased in the femora of HFD-4W and HFD-16W mice (Figure 3C and D). Notably, the proportion of increased TRAP expression after 4 weeks of HFD feeding was higher than that after 16 weeks, indicating significant enhancement of bone resorption activity in the early phase of T2D. Macrophages play various roles in bone homeostasis, such as mediating osteoclastogenesis and immune regulation 29. Enlightened by the literature of others, we stained femoral macrophages of T2D mice. F4/80 expression was significantly elevated in the femora of HFD-4W and HFD-16W mice, which was more prominent at 4 weeks of HFD feeding, indicating evident changes in immune cells at the early T2D progression (Figure 3E and F). Inflammation, especially chronic inflammation, plays a significant role in the pathogenesis of osteoporosis, which leads to an imbalance in bone remodeling, favoring bone resorption over bone formation 34. In specific, as a chronic inflammatory disease, T2D is characterized by elevated levels of multiple cytokines 35. As a major proinflammatory mediator, tumor necrosis factor-α (TNF-α) is thought to interfere with bone homeostasis by inhibiting bone formation by osteoblasts and promoting bone resorption by osteoclasts 27, 36-38. As to the involvement of TNF-α in diabetic bone disease, IF staining results demonstrated that the expression of TNF-α was increased in the femora of HFD-4W and HFD-16W mice (Figure 3G and H). Intriguingly, there was a higher proportion of TNF-α expression increases in HFD-4W mice than in HFD-16W mice, suggesting that inflammation is likely to be intimately involved in the occurrence and development of osteoporosis in the early phase of T2D. To further understand the bone changes under T2D, we examined procollagen type 1 N-terminal propeptide (P1NP) and C-terminal cross-linked telopeptide 1 (CTX1) in serum, which respectively reflects the bone formation rate and the bone resorption rate. We found that HFD-induced T2D mice had significantly decreased concentrations of P1NP and increased CTX1 compared to control mice (Figure 3I-J), indicating an altered bone turnover in T2D mice. Together, these results highlight alterations of bone remodeling and immune-related inflammation in HFD-induced T2D mice.

### Dysregulated bone marrow gene expression profiles along the T2D disease progression

**Figure 4:**
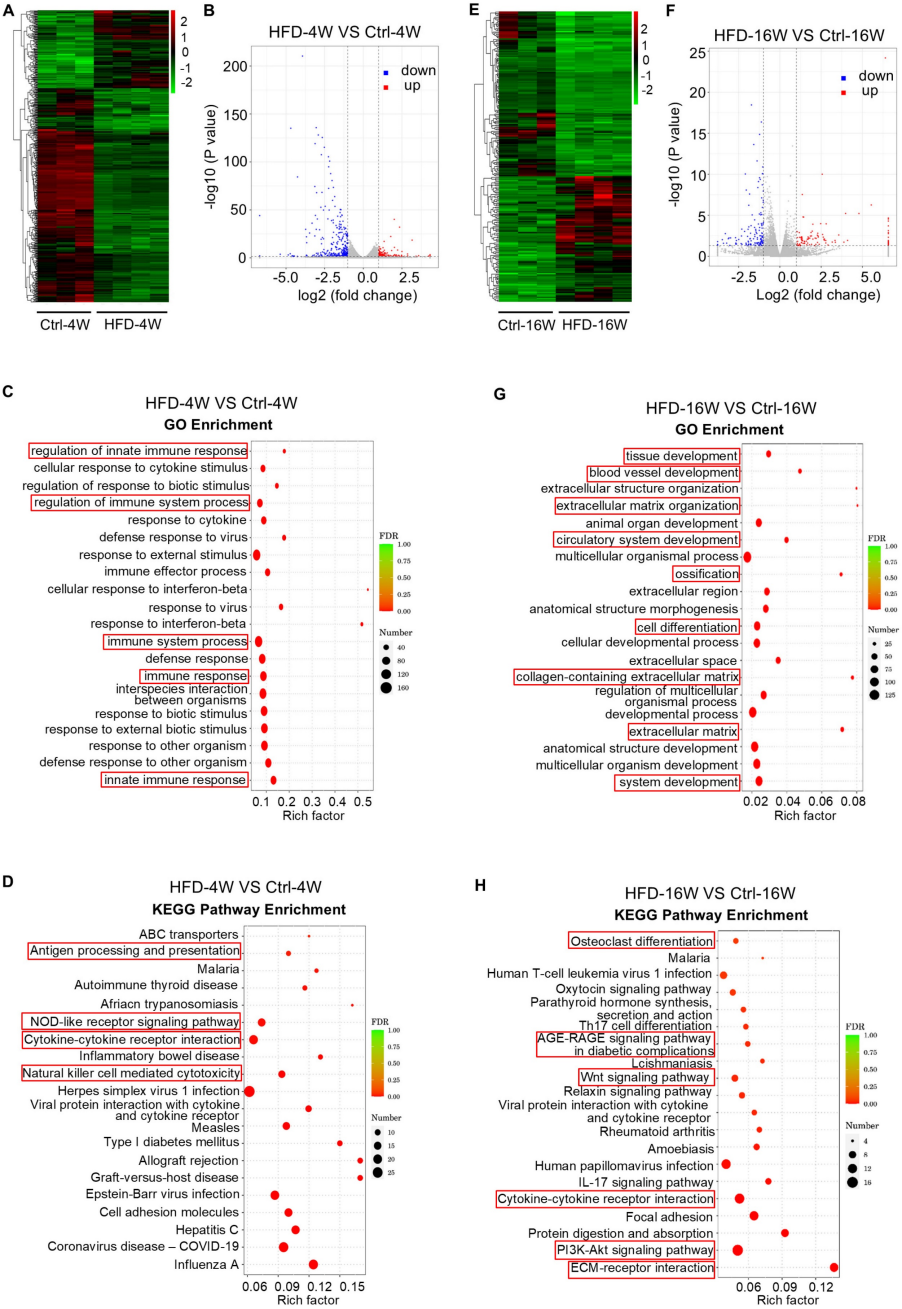
** Dysregulated bone marrow gene expression profiles in different time points of type 2 diabetes (T2D) mice.** (A) Hierarchical clustering heatmap of differentially expressed genes (DEGs) (Fold change > 2 and *P* value < 0.05) between high fat diet (HFD)-feeding for 4 weeks (HFD-4W) and control (Ctrl-4W) mice bone marrow, with gene expression abundance being Z-score normalized. Rows represent DEGs and columns represent individual replicates. (B) Volcano plot showing significantly downregulated (blue dots) and upregulated (red dots) genes in HFD-4W mice bone marrow, compared to Ctrl-4W mice. (C) Gene ontology (GO) enrichment analysis of the DEGs between HFD-4W and Ctrl-4W mice bone marrow. The Y-axis represents GO terms and the X-axis represents rich factor. The size of the bubble represents number of enriched DEGs and the color of the bubble represents enrichment significance. (D) Kyoto Encyclopedia of Genes and Genomes (KEGG) enrichment analysis of the DEGs between HFD-4W and Ctrl-4W mice bone marrow. The Y-axis represents KEGG pathways and the X-axis represents rich factor. The size of the bubble represents number of enriched DEGs and the color of the bubble represents enrichment significance. (E) Hierarchical clustering heatmap of DEGs (Fold change > 2 and *P* value < 0.05) between HFD-feeding for 16 weeks (HFD-16W) and control (Ctrl-16W) mice, with gene expression abundance being Z-score normalized. Rows represent DEGs and columns represent individual replicates. (F) Volcano plot showing significantly downregulated (blue dots) and upregulated (red dots) genes in HFD-16W mice bone marrow, compared to Ctrl-16W. (G) GO enrichment analysis of the DEGs between HFD-16W and Ctrl-16W mice bone marrow. The Y-axis represents GO terms and the X-axis represents rich factor. The size of the bubble represents number of enriched DEGs and the color of the bubble represents enrichment significance. (H) KEGG enrichment analysis of the DEGs between HFD-16W and Ctrl-16W mice bone marrow. The Y-axis represents KEGG pathways and the X-axis represents rich factor. The size of the bubble represents number of enriched DEGs and the color of the bubble represents enrichment significance.

To obtain a comprehensive understanding of the gene expression patterns and dynamics within bone tissues during the progression of T2D osteopathy, transcriptome sequencing was performed on the bone marrow of HFD-induced T2D mice at 4 and 16 weeks by adopting the commonly used method of bone marrow flush. Compared with the control group, a total of 528 differentially expressed genes (DEGs) were identified in the bone marrow gene expression profiles of mice fed with HFD for 4 weeks, among which 140 genes were up-regulated and 388 genes were down-regulated (Figure 4A and B). Further functional enrichment analysis was performed for these DEGs. Gene Ontology (GO) enrichment analysis showed that the top 20 enriched items of these DEGs were mainly focused on immune-related items, such as “regulation of innate immune response”, “regulation of immune system process”, “immune system process”, “immune response” and “innate immune response” (Figure 4C).

In addition, we performed the Kyoto Encyclopedia of Genes and Genomes (KEGG) enrichment analysis of the biological pathways involved in these DEGs. The top 20 pathways enriched by these DEGs were mainly involved in “Antigen processing and presentation”, “NOD-like receptor signaling pathway”, “Cytokine-cytokine receptor interaction” and “Natural killer cell-mediated cytotoxicity” (Figure 4D). According to the KEGG enrichment results of DEGs, among the top 30 pathways with the most significant enrichment screened, there were also enriched pathways related to osteoclast differentiation (Figure S1). These results suggest that 4 weeks of HFD feeding significantly affects the immune microenvironment within the bone, which underlies the disturbance of bone homeostasis in the early phase.

On the other hand, HFD feeding for 16 weeks altered the expression of 234 genes, of which 101 genes were up-regulated and 133 genes were down-regulated (Figure 4E and F). We then focused on these DEGs and carried out functional analysis. GO enrichment analysis revealed that the top 20 items enriched for these DEGs include “extracellular matrix organization”, “ossification”, “cell differentiation”, “collagen-containing extracellular matrix” and “extracellular matrix” (Figure 4G). Further KEGG enrichment analysis showed that these DEGs were associated with “Osteoclast differentiation”, “Oxytocin signaling pathway”, “Parathyroid hormone synthesis, secretion and action”, “AGE-RAGE signaling pathway in diabetic complications”, “Wnt signaling pathway”, “Relaxin signaling pathway”, “PI3K-Akt signaling pathway” and “ECM-receptor interaction” (Figure 4H). These findings indicate that long-term HFD feeding induces noticeable changes in genes in bone formation and resorption, involving various hormones and signaling pathways related to bone homeostasis.

### BMMSCs are critically involved during the HFD-induced T2D progression

**Figure 5:**
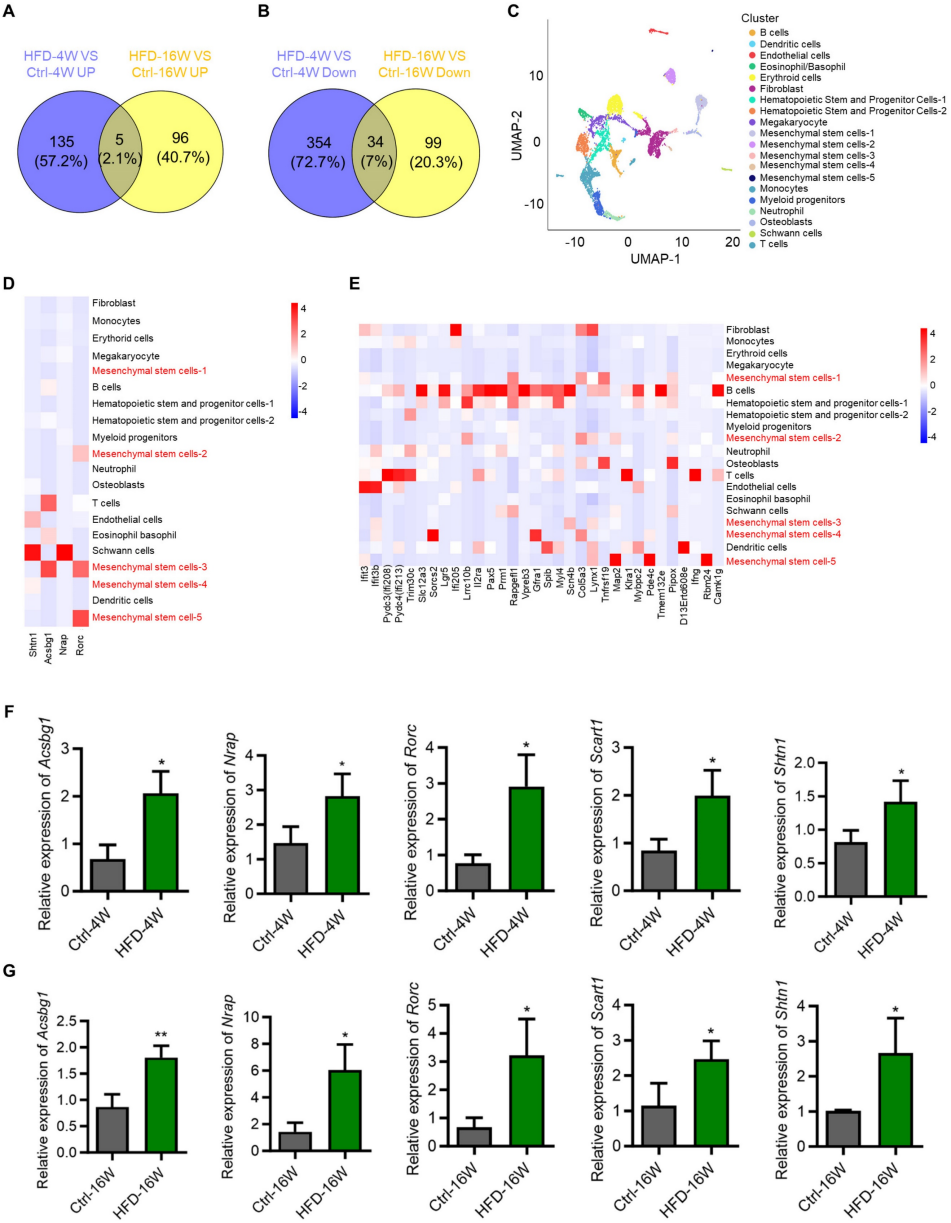
** Bone marrow mesenchymal stem cells (BMMSCs) are critically involved in the disease progression of type 2 diabetes (T2D) mice.** (A) Venn diagram showing overlapping genes between up-regulated genes in HFD-feeding for 4 weeks (HFD-4W) versus control (Ctrl-4W) mice and up-regulated genes in HFD-feeding for 16 weeks (HFD-16W) versus Ctrl-16W mice. (B) Venn diagram showing overlapping genes between down-regulated genes in HFD-4W versus Ctrl-4W mice and down-regulated genes in HFD-16W versus Ctrl-16W mice. (C) Uniform manifold approximation and projection (UMAP) plot showing unbiased clustering of bone marrow cells. General identity of each cell cluster is defined on the right. (D) Heatmap showing the expression of up-regulated overlapping genes of panel A in different bone marrow cell clusters. (E) Heatmap showing the expression of down-regulated overlapping genes of panel B in different bone marrow cell clusters. (F) Quantitative real-time polymerase chain reaction (qRT-PCR) analysis of up-regulated overlapping genes of panel A of *Acyl-CoA synthetase bubblegum family member 1* (*Acsbg1*), *Nebulin-related anchoring protein* (*Nrap*), *RAR-related orphan receptor gamma* (*Rorc*), *Scavenger receptor family member expressed on T cells 1* (*Scart1*) and *Shootin 1* (*Shtn1*) in BMMSCs from HFD-4W and Ctrl-4W mice. *β-actin* (*Actb*) was used as a reference gene, and quantification of fold changes was performed over the Ctrl group. *N* = 3 per group. (G) qRT-PCR analysis of up-regulated overlapping genes of panel A of *Acsbg1*,* Nrap*, *Rorc*, *Scart1* and *Shtn1* in BMMSCs from HFD-16W and Ctrl-16W mice. *β-actin* (*Actb*) was used as a reference gene. *N* = 3 per group. Mean ± standard deviation (SD). *, *P* <0.05; **, *P* <0.01. Student's two-tailed unpaired *t* tests.

Considering different changes in the transcriptome between HFD-4W and HFD-16W, we intended to explore genes with consistent changes throughout the different periods of HFD-induced T2D pathogenesis, which may play an important role in the development of T2D-associated osteoporosis. Accordingly, we performed Venn analysis of the DEGs between HFD-4W and Ctrl-4W, as well as HFD-16W and Ctrl-16W, and screened 39 common DEGs, of which 5 genes were up-regulated (Figure 5A) and 34 were down-regulated (Figure 5B). To further clarify the cell types within the bone tissue expressing these genes, we performed a bioinformatics analysis using a scRNA-seq dataset of bone and bone marrow cells from a previous study 27. After undergoing strict quality control, we gathered gene expression data for clustering analyses from a total of 7,497 cells. This analysis unveiled 20 distinct cell populations, which were visualized as uniform manifold approximation and projection (UMAP) embeddings (Figure 5C). Particularly, five clusters highly expressed MSC markers. We further analyzed the expression of the 39 overlapping genes in the various types of bone and bone marrow-derived cells. Notably, among the commonly up-regulated genes (only 4 of 5 detected in the scRNA-seq dataset), 2 were predominantly expressed by BMMSCs and 2 were mainly expressed by Schwann cells (Figure 5D). Additionally, among the commonly down-regulated genes, 8 were mainly expressed by BMMSCs (Figure 5E). Besides, the number of genes mainly expressed by B cells and T cells was relatively high as well (Figure 5E). To verify the bioinformatic analysis results, we selected the overlapping up-regulated genes as the targets of interest, and expression of the 5 genes (despite 1 not being detected in the scRNA-seq dataset) in BMMSCs from HFD-4W, HFD-16W, and control mice were examined by quantitative real-time polymerase chain reaction (qRT-PCR) analysis. Results showed that expression levels of *Acyl-CoA synthetase bubblegum family member 1* (*Acsbg1*), *Nebulin-related anchoring protein* (*Nrap*), *RAR-related orphan receptor gamma* (*Rorc*), *Scavenger receptor family member expressed on T cells 1* (*Scart1*) and *Shootin 1* (*Shtn1*) had significantly up-regulated tendency in BMMSCs from HFD-fed mice (Figure 5F and G), which was consistent with the sequencing results. These results suggest that BMMSCs are likely to be the important cells involved in the ongoing pathogenesis of bone disease in T2D, while immune cells and other cells are also involved.

### Dysfunction of BMMSCs in different time points of HFD-induced T2D mice

**Figure 6:**
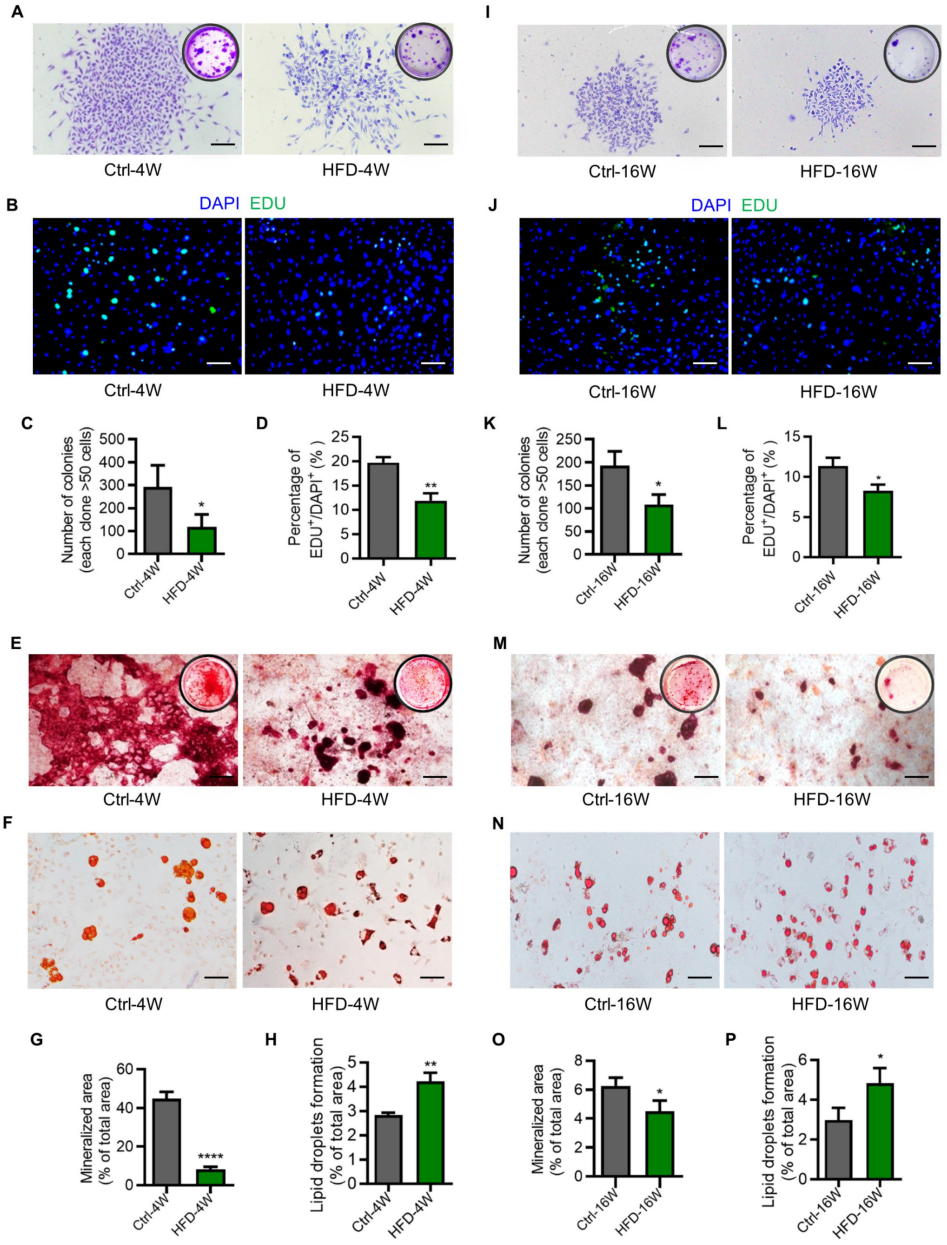
** Dysfunction of bone marrow mesenchymal stem cells (BMMSCs) in different time points of type 2 diabetes (T2D) mice.** (A) Representative crystal violet staining images demonstrating the colony forming ability of BMMSCs derived from HFD-feeding for 4 weeks (HFD-4W) and control (Ctrl-4W) mice. Scale bars, 100 µm. (B) Representative 5-ethynyl-2'-deoxyuridine (EDU) staining images demonstrating the proliferation potential of BMMSCs derived from HFD-4W and Ctrl-4W mice. The green fluorescence indicates EDU-positive proliferating cells, and the blue represents the DAPI-stained nuclei. Scale bars, 100 µm. (C) Corresponding quantification of the number of colonies with over 50 cells in panel A. *N* = 3 per group. (D) Corresponding quantification showing the percentage of EDU^+^/DAPI^+^ area in panel B. *N* = 3 per group. (E) Representative alizarin red S staining images demonstrating the osteogenic differentiation potential of BMMSCs derived from HFD-4W and Ctrl-4W mice. Scale bars, 100 µm. (F) Representative oil red O staining images demonstrating the adipogenic differentiation potential of BMMSCs derived from HFD-4W and Ctrl-4W mice. Scale bars, 100 µm. (G) Corresponding quantification of the percentage of mineralized area in panel E. *N* = 3 per group. (H) Corresponding quantification of the percentage of lipid droplets formation area in panel F. *N* = 3 per group. (I) Representative crystal violet staining images demonstrating the colony forming ability of BMMSCs derived from the HFD-feeding for 16 weeks (HFD-16W) and control (Ctrl-16W) mice. Scale bars, 100 µm. (J) Representative EDU staining images demonstrating the proliferation potential of BMMSCs derived from HFD-16W and Ctrl-16W mice. Scale bars, 100 µm. (K) Corresponding quantification of the number of colonies with over 50 cells in panel I. *N* = 3 per group. (L) Corresponding quantification showing the percentage of EDU^+^/DAPI^+^ area in panel J. *N* = 3 per group. (M) Representative alizarin red S staining images demonstrating the osteogenic differentiation potential of BMMSCs derived from the HFD-16W and Ctrl-16W mice. Scale bars, 500 µm. (N) Representative oil red O staining images demonstrating the adipogenic differentiation potential of BMMSCs derived from HFD-16W and Ctrl-16W mice. Scale bars, 100 µm. (O) Corresponding quantification of the percentage of mineralized area in panel M. *N* = 3 per group. (P) Corresponding quantification of the percentage of lipid droplets formation area in panel N. *N* = 3 per group. Mean ± standard deviation (SD). *, *P* <0.05; **, *P* <0.01; ***, *P* < 0.001. Student's two-tailed unpaired *t* tests.

As stated above, as precursors of the osteogenic lineage and modulators of various bone cells, BMMSCs have been acknowledged as significant contributors to bone homeostasis 39, 40. While previous studies have indicated that BMMSCs derived from diabetic individuals exhibit impaired functions, their functional changes along the disease progression of T2D remain largely unknown 17, 41. To provide a better understanding of this issue, we isolated BMMSCs from HFD-4W and HFD-16W mice, as well as their control mice, and evaluated their ability of colony-forming, proliferation, and differentiation potential. Results showed that BMMSCs derived from both HFD-4W and HFD-16W mice exhibited significantly reduced clonogenicity compared to the control group, as demonstrated by crystal violet staining and quantitative analysis (Figure 6A, C, I and K). The number of proliferative 5-ethynyl-2'-deoxyuridine (EDU)-positive cells in the HFD groups were further significantly decreased compared to the control groups (Figure 6B, D, J and L).

Additionally, the clonogenic and proliferative capacity of BMMSCs from mice modeled for 16 weeks was lower than that of the control mice with 4 weeks of modeling (Figure 6A-D, I-L). Similarly, BMMSCs derived from both HFD-4W and HFD-16W mice showed dramatic declines in osteogenesis, as demonstrated by alizarin red S staining and quantitative analysis (Figure 6E, G, M and O).

Besides, BMMSCs from mice that were modeled for 16 weeks exhibited a lower osteogenic differentiation capacity compared to the control mice at 4 weeks of modeling (Figure 6E, G, M and O). Moreover, the adipogenic differentiation ability of BMMSCs from HFD-4W and HFD-16W mice was higher than those from control mice (Figure 6F, H, N and P). Collectively, these data indicated that functional abnormalities in BMMSCs appear early in T2D, which may represent a significant cellular basis for diabetes-induced osteoporosis.

## Discussion

T2D is a prevalent chronic metabolic disorder associated with a range of complications affecting multiple organ systems, including bone. Notably, epidemiological studies have demonstrated that individuals with T2D have an elevated risk of fractures 42, 43. Animal research has also shown that T2D compromises skeletal strength and quality 16. Nevertheless, the exact impact of T2D on bone, particularly in the early phase, remains to be elucidated. There is a limited comprehensive analysis of the T2D bone phenotype by investigating a verified T2D mouse model with different time points along the disease progression. In this study, we established the T2D mouse model induced by different durations of HFD feeding, allowing us to closely examine the bone pathology associated with T2D progression. We observed osteoporotic symptoms, particularly in the trabecular bone, even in the early phase of T2D, indicating that bone alterations occur during the onset of the disease. We found that T2D led to decreased osteogenic activity, increased osteoclastic activity, and an elevated level of immune-related proinflammatory factor, particularly in the early phase. Furthermore, transcriptomic sequencing analysis revealed substantial changes in the gene expression profiles of bone throughout the progression of T2D, with distinct characteristics at different time points. Importantly, scRNA-seq analysis and functional testing revealed early functional abnormalities with dysregulated molecular signature of BMMSCs. These findings shed light on HFD-induced T2D bone lesions, especially at the onset of metabolic alterations, and highlight the importance of early interventions to prevent or mitigate T2D-associated bone complications. The insights gained from examining alterations in gene expression profiles across the T2D progression provide a foundation for future research endeavors aimed at developing targeted treatment strategies specifically tailored for T2D-related bone diseases.

The development of sequencing technologies has provided available tools for comprehensive analysis of molecular biology changes in bone tissue under pathological conditions 44-46. In this study, transcriptome sequencing has helped us gain an understanding of the gene expression patterns and dynamics within the bone tissue during the progression of T2D osteopathy. In the early phase of T2D, marked alterations in gene expression are primarily associated with immunity, indicating that the immune microenvironment within the bone is significantly affected by 4 weeks of HFD feeding, potentially contributing to the disturbance of bone homeostasis. Considering the close relationship between immunity and bone 47, 48, and the frequent occurrence of immune dysfunction in T2D 49, further analysis of immune-mediated T2D osteoporosis will help to clarify potential therapeutic targets. Notably, overlapping down-regulated genes along the T2D progression are predominantly expressed in B cells. Interestingly, B lymphocytes are emerging players in promoting inflammation associated with insulin resistance and T2D by producing IgG antibodies, which in turn enhance local TNF-α and interferon-gamma (IFN-γ) production from macrophages and T cells 50. Intriguingly, B cells themselves show a 52% reduction in the bone marrow of mice maintained on HFD for 7 months, which might be related to our detected down-regulation of B cell genes in T2D 51. The detailed function of B cells in T2D osteoporosis will be investigated in our future work. Moreover, long-term HFD feeding induces substantial changes of expression in genes associated with bone formation and resorption, involving various hormones and signaling pathways crucial for maintaining bone homeostasis, such as oxytocin 52, 53, parathyroid hormone 54, 55, Wnt signaling pathway 56, and relaxin 57, 58. This result indicates that the osteoporosis caused by long-term T2D may be the result of the combined action of multiple mechanisms. Overall, the transcriptomic analysis of bone marrow gene expression profiles in T2D mice at different time points highlights the dynamic nature of molecular changes occurring in the bone microenvironment during T2D development and provides valuable insight into mechanisms underlying T2D-induced bone complications. Further exploration of these identified DEGs and pathways may lead to the development of targeted therapeutic approaches for managing bone complications in T2D patients.

Previous studies have demonstrated that bone formation by osteoblasts and bone resorption by osteoclasts maintain a dynamic balance under physiological conditions 59, 60. Osteoporosis is a disorder of bone metabolism often caused by an imbalance between bone resorption and bone formation 61. Notably, although studies have found that T2D can lead to a decrease in osteogenic activity, there is still controversy regarding changes in osteoclastic activity 16, 62, 63. Park *et al*. reported that bone turnover was reduced in T2D with reduced bone resorption 63, while Guo *et al*. reported that the bone mass loss in mice exposed to HFD was mainly due to increased osteoclast formation and bone resorption activity 62. In the present study, we detected reduced bone formation and increased bone resorption in the femora of T2D mice, with these phenotypic changes being more pronounced in the early phase. For the immune-related items, F4/80 staining of femoral macrophages showed the proportion of increased F4/80 expression after 4 weeks of HFD feeding was higher than that after 16 weeks of HFD feeding, indicating that changes in immune cells might also be more evident in the early phase of T2D, which will be continued to be investigated in future works. In addition, we observed a significant increase in TNF-α expression in the femora of both HFD-4W and HFD-16W mice. TNF-α has been shown to interfere with bone homeostasis by inhibiting osteoblast-mediated bone formation and promoting osteoclast-mediated bone resorption 36. Indeed, T2D is characterized by elevated levels of proinflammatory cytokines, such as TNF-α, which mediate the occurrence of multiple T2D complications 35, 64. Interestingly, the proportion of increased TNF-α expression was higher in HFD-4W mice than in HFD-16W mice, indicating that inflammation may play an early important role in the development of T2D-induced osteoporosis, consistent with our transcriptomic analysis. These findings suggest that in the early phase of T2D, not only is already osteoporosis occurring, but also the alterations of bone remodeling are more vigorous, highlighting the importance of early intervention.

Combined analysis of the mouse bone marrow transcriptome and scRNA-seq data revealed a close relationship between BMMSCs and the development of T2D-associated bone disease. This finding is consistent with published studies, suggesting that dysfunction of BMMSCs may be a core factor in T2D-related osteoporosis 17, 43. Notably, the bone marrow flush methodology collects MSCs mostly from the bone marrow cavity rather than the periosteum or the endosteum, whereas stem cell populations residing in different locations of bone show heterogeneity and may demonstrate discrepancies in response to extrinsic stimuli 65-67. Future research is in demand to investigate in detail the BMMSC response to T2D in a spatially dependent manner. Furthermore, *in vitro* functional experiments revealed decreased colony-forming, proliferation, and osteogenic differentiation abilities of BMMSCs in T2D mice, along with enhanced adipogenic differentiation ability. These results not only align with previous findings demonstrating impaired proliferation and survival, diminished osteogenic differentiation capacity, and increased adipogenic differentiation capacity of BMMSCs from diabetic animals 68, 69, but also indicate early emergence of functional disorders of BMMSCs in T2D, potentially serving as a substantial cellular foundation for T2D-induced osteoporosis. Furthermore, T2D has been reported to alter the secretome composition and impair the angiogenic properties of BMMSCs 70, 71. The impairment of functions of BMMSCs has been attributed to the combined effects of hyperglycemia, advanced glycation end products, oxidative stress, and inflammation induced by T2D, which synergistically result in a pathological niche 72. Molecularly, we have performed a brief exploration of the potential mechanisms underlying the osteoporotic phenotypes by examining five genes of interest (*Acsbg1, Nrap, Rorc, Scart1, Shtn1*) in BMMSCs. Particularly, expression changes of these genes at the early stage have been identified and will be focused on as mechanistic targets in future studies. Specifically, *Acsbg1* encodes a very long-chain acyl-CoA synthetase, which serves as a checkpoint of lipid metabolism by regulating mitochondrial fitness 73*.* In addition, *Nrap* encodes an actin-associated ankyrin, while *Scart1* encodes a scavenger receptor predicted to be involved in endocytosis 74, 75*. Rorc*, intriguingly, encodes a member of the nuclear orphan receptor family and performs critical regulatory functions in cell proliferation, and *Shtn1* encodes a protein mediating the mechanical coupling between F-actin retrograde flow and cell adhesions as a clutch molecule 76, 77. Although the function of these genes in BMMSCs and bone is unclear, further elucidation of the molecular mechanisms underlying BMMSC impairment and their regulatory pathways will aid in establishing effective strategies for treating diabetic osteoporosis. Moreover, as an important “seed cell” for regenerative medicine, the impact of diabetic microenvironment on BMMSCs should also draw attention to their translational applications.

In conclusion, this study sheds light on T2D-induced bone lesion phenotype, especially at the early phase, and uncovers the significant alterations in the gene expression profile during T2D progression. Moreover, it provides novel insights into the functional changes occurring in bone cells, including a decreased osteogenic activity, an increased osteoclastic activity, and an elevated immune-related proinflammatory factor level, highlighting the crucial role of BMMSCs in T2D osteoporosis. These findings will aid in the development of effective treatment strategies for T2D-related bone complications.

## Materials and Methods

### Animals

All animal experiments were performed following the ARRIVE guidelines, in compliance with the relevant laws and ethical regulations, following the Guidelines of Intramural Animal Use and Care Committees of The Fourth Military Medical University, and were approved by the Institutional Review Board of The Fourth Military Medical University (No. kq-2023-045). C57BL/6 mice were purchased from the Laboratory Animal Center of the Fourth Military Medical University. All mice used in this study were male and maintained at 24°C in a 12/12-h light/dark cycle in a specific pathogen-free facility and given free access to food and water. The sample number N represents individual mouse numbers. Sample sizes were determined according to preliminary experiments. All animals were randomly assigned to cohorts when used with confounders not being controlled 78. Researchers were blind to the group allocations during the study.

### *In vivo* modeling

To induce T2D models, 5-week-old male C57BL/6J mice were fed with an HFD (60 kcal% from fat, D12492, Research Diets, USA) diet for 4 weeks and 16 weeks, respectively. Control mice were fed with a normal chow diet (NCD) and were set as the Ctrl group. T2D mice used in the experiments were selected based on their blood glucose and glucose tolerance tests (GTT).

### Blood glucose detection

Blood glucose tests were performed using a glucometer (Roche, Germany). For fasting blood glucose, the mice were fasted for 16h and the blood was collected from the tail vein for testing. For GTT, the mice were fasted for 16h and intraperitoneally injected with 20% glucose (Sigma-Aldrich, USA) at a dose of 2 g/kg body weight. At 0, 15, 30, 60, 90, and 120 min, the blood samples were collected from the tail vein, and the blood glucose values were measured 79. For insulin tolerance tests (ITT), after fasting for 6h, the mice were intraperitoneally injected with human insulin (Novo Nordisk AS, Denmark) at a dose of 1 IU/kg body weight. The blood samples were collected from the tail vein at 0, 15, 30, 60, 90, and 120 min after injection to detect the blood glucose 80.

### Micro-CT analysis

A desktop micro-CT system (eXplore Locus SP, GE Healthcare, USA) was employed, as previously used 81. The mice were sacrificed, and the left femora were removed and fixed overnight in 4% paraformaldehyde (PFA) (Biosharp, China) at 4°C. Then, the femora were scanned with the micro-CT system at a voltage of 80kV, a current of 80μA, and a resolution of 8μm. The three-dimensional image was established and the region of interest (ROI) of distal femur metaphysis was selected for each sample. The region of interest (ROI) of cortical bone was defined from 3.3mm away from the epiphysis along the midshaft, and with a thickness of 0.5mm. The ROI of the trabecular bone was defined from 1mm to 1.7mm away from the epiphysis. Micview V2.1.2 software was used to analyze the corresponding parameters of ROI 82.

### Enzyme-linked immunosorbent assay (ELISA)

Mouse serum was collected, and HbA1c percentages were detected using a murine ELISA kit according to the manufacturer's instructions (ENZO-2, USA). Insulin levels were detected using the Mouse Insulin ELlSA Kit (Ultrasensitive) according to the manufacturer's instructions (Beyotime, China) 83. Serum P1NP and CTX1 levels were also detected using commercial ELISA kits (Fankew, China).

### Transcriptome sequencing

The femora and tibiae of mice were removed at sacrifice, and the bone marrow was flushed out with normal saline. According to our and other's reports, this method collects whole bone marrow cells, including MSCs mostly from the bone marrow cavity 31, 40, 65, 81. The bone marrow was collected into a centrifuge tube, lysed with erythrocyte lysis buffer (Coollaber, China) on ice for 2 min, and centrifuged at 500g for 5 min. After removing the supernatant sediment, the remaining cells were resuspended with normal saline, centrifuged at 500 g for 5 min, and the deposited cells were collected for RNA extraction. The total RNA of each sample was extracted using the RNeasy Mini Kit (Qiagen, Germany). The purity and integrity of RNA were checked, and 1μg qualified total RNA from each sample was used to construct the transcriptome sequencing library. The sequencing libraries were generated according to the NEBNext®Ultra™RNA Library Preparation Kit (NEB, USA) operating guidelines, and the index codes were added to the attribute sequence of each sample. The mRNA was purified from total RNA using Poly-Toligo-magnetic beads, and total RNA samples were fragmented at a high temperature in NEBNext first chain synthesis reaction buffer (5X) with divalent cations. First-strand cDNA was synthesized, followed by second-strand cDNA synthesis using DNA polymerase I and RNase H. A NEBNext Adaptor with a hairpin loop structure was conjugated to the 3'-end adenylated DNA fragment. The AMPure XP system (Beckman Coulter, Beverly, USA) was used to screen 350 bp cDNA fragments. Then, 3µL of USER Enzyme (NEB, USA) was mixed with the selected cDNA, reacted at 37°C for 15 min, and ligated at 95°C for 5 min before PCR. Then, PCR was performed with Phusion High-Fidelity DNA polymerase, Universal PCR primers, and Index (X) Primer. Finally, PCR products were purified by the AMPure XP system, and library quality was assessed on the Agilent Bioanalyzer 2100 system and quantified by Qubit 2.0 Fluorometer (Invitrogen, Carlsbad, CA, USA). The clustering of the index-coded samples was performed on a cBot Cluster Generation System according to the instructions of the TruSeq PE Cluster Kit v3-cBot-HS (Illumia). Sequencing was performed and paired-end reads were generated on the llumina Hiseq platform. Firstly, the raw data were filtered and the clean data were aligned to the species reference genome using the Hisat2 tool software 84. The expression levels of genes were calculated. DESeq was used to analyze the difference in gene expression. |log_2_FoldChange|>1 and *P* value<0.05 were set as a condition for screening DEGs 85. Enrichment analysis of DEGs was further performed based on GO and KEGG databases. The importance value of each pathway was measured using degree centrality. *P* values, at the same time, were adjusted by the FDR method 86. Venn analysis was performed at the Venny 2.1 website (https://bioinfogp.cnb.csic.es/ tools/venny/index.html).

### Re-analysis of scRNA-seq data

Published scRNA-seq data were downloaded from the Gene Expression Omnibus (GEO). Single-cell gene expression matrices were generated by aligning themm10 reference genome with the R language software in CellRanger software (version 3.1.0), which were converted into Seurat objects using the Seurat R package (version 3.0.1). The Seurat R package allows us to easily explore QC metrics and filter cells. All cells were removed that had either more than 20,000 UMIs, over 4,000 or below 500 expressed genes, or over 10% UMIs derived from the mitochondrial genome. Dimensionality reduction for visualizing single-cell data was performed using UMAP analysis. Clusters were identified using the Seurat function. Data structures were visualized and explored with UMAP to show deviations between different cell types. Next, differentially expressed genes were determined using the Wilcoxon test implemented in the “FindAllMarkers” function, which was considered significant with an average natural logarithm (fold change) of at least 0.25 and a *P* value lower than 0.01 87. For expression heatmap analysis of DEGs in single-cell data. Firstly, the expression data files of the genes of interest in the transcriptome data were screened by Venn analysis. The expression data file was then read using the 'read_excel' function in R, and the heatmap package (Version 1.0.12) was used to generate the heatmap.

### IF staining

The freshly dissected femora were collected and fixed in 4% PFA (Biosharp, China) solution for 4 h at 4°C. The femora were decalcified by 17% EDTA (pH=7.2-7.4) (Proandy, China), dehydrated by 30% sucrose (Sigma-Aldrich, USA), and then embedded in OCT (Leica, Germany). Sagittal serial sections of 20µm were prepared with freezing microtome (CM1950, Leica, Germany). The frozen sections were air-dried for 1 h at room temperature and permeabilized with 0.3% Triton X-100 (Sigma-Aldrich, USA) for 20 min at room temperature. After washing with PBS 3 times, the sections were blocked with goat serum (BOSTER, China) for 30 min at room temperature, and incubated with primary antibodies at 4°C overnight in the dark. The primary antibodies were anti-RUNX2 (#12556, Cell Signaling, USA, diluted 1:400), anti-TRAP (ab191406, Abcam, UK, diluted 1:100), anti-F4/80 (123120, BioLegend, USA, diluted 1:200) and anti-TNF-α (sc-52746, Santa Cruz Biotechnology, USA, diluted 1:400). TRAP IF staining was selected as an alternative to traditional TRAP staining according to our previous report 88. After washing with PBS3 times, the sections were incubated with fluorescently labeled secondary antibodies, Alexa Flour®488 Donkey anti-Rabbit IgG (ab150073, Abcam, UK, diluted 1:200), Cy3™ AffiniPure Goat Anti-Rabbit IgG (33108ES60, Yeasen, China, diluted 1:200), and Alexa Flour®594 Goat Anti-Mouse IgG (33212ES60, Yeasen, China, diluted 1:200), for 1 h at room temperature. After washing with PBS 3 times, the slides were mounted with a Mounting Medium With DAPI-Aqueous Fluoroshield (Abcam, UK). Photographs were taken by a confocal microscope (FV1000, Olympus, Japan) and analyzed using the ImageJ software. Quantification of IF staining was performed based on our published methods 89, 90.

### Isolation and culture of murine BMMSCs

Isolation and culture of mouse BMMSCs were performed according to the methods described in the previous studies 31, 40, 81. In brief, the mice were euthanized, and the hind limbs were dissected and removed of soft tissues under aseptic conditions. The bone marrow cavities of the femora and tibiae were rinsed with growth medium, α-MEM supplemented with 20% fetal bovine serum (FBS; Corning, USA) and 1% penicillin and streptomycin (Procell, China). The cell suspension was collected and filtered by a 70 μm nylon filter (BD Falcon, USA). The cells were centrifuged at 500 g for 5 min at 4°C, the supernatant was discarded, and the cell precipitate was resuspended in a growth medium and seeded in 10 cm tissue culture dishes (NEST, USA). After being incubated for 24 h at 37°C in a humidified atmosphere of 5% CO_2_, non-adherent cells were removed by rinsing with PBS. The medium was changed every 2 days. When the cells were 90% confluent, they were digested with 1 X TrypLE™ express (Invitrogen, USA) and passaged.

### Colony forming and crystal violet staining

Primary mouse bone marrow cells were isolated, counted, and seeded at a density of 2 × 10^4^ per dish in 6-cm diameter Petri dishes (NEST, USA). The medium was changed every 3 days. On day 14, after discarding the supernatant, the cells were washed 3 times with PBS and fixed with 4% PFA (Biosharp, China) for 20 min at room temperature. After washing with PBS 3 times, the cells were stained with 0.1% crystal violet solution (Solarbio, China). Colonies with over 50 cells were included for calculation. Photographs were taken using an inverted optical microscope (Olympus, Japan) and a camera (Canon, Japan).

### EDU proliferation assay

BMMSCs at the second passage were seeded in 24-well plates (Corning, USA) at 1×10^4^ cells per well and incubated for 24 h. Cells were then treated with EDU at a working concentration of 10μM in 200μL of culture medium for 48 h. Afterward, the cells were detected using a kFluor488-EdU Cell Proliferation Test Kit (KeyGEN Biotech, China) according to the manufacturer's instructions.

### Osteogenic differentiation and alizarin red S staining

BMMSCs at the second passage were induced to osteogenic differentiation. The cells were seeded in 12-well plates at a density of 2 × 10^5^ cells/well. When the cell growth reached about 70% confluence, the growth medium was changed into osteogenic differentiation induction medium, α-MEM medium containing 20% FBS (Corning, USA), 10nM dexamethasone (Sigma-Aldrich, USA), 100 μg/ml ascorbic acid (MP Biomedicals, USA) and 2mm β-glycerophosphoric acid (Sigma-Aldrich, USA), which was replaced every 3 days. After 14 days of treatment, alizarin red S (Sigma-Aldrich, USA) staining was performed to determine the mineralization 91. Photographs were taken using an inverted optical microscope (Olympus, Japan) and a camera (Canon, Japan). The percentage of mineralized area was determined using the ImageJ software.

### Adipogenic differentiation and oil red O (ORO) staining

BMMSCs at the second generation were induced to adipogenic differentiation. The cells were seeded in 12-well plates at a density of 2 ×10^5^ cells/well. When the cell growth reached about 90% confluence, the growth medium was changed into adipogenic differentiation induction medium, α-MEM containing 20% FBS (Corning, USA), 60mm indomethacin (MCE, USA), 0.5mm isobutyl methylxanthine (MCE, USA) and 0.5μM dexamethasone (Sigma-Aldrich, USA), which was replaced every two days. After 14 days of treatment, ORO (Aladdin, China) staining was used to detect the formation of lipid droplets 92. Photographs were taken using an inverted optical microscope (Olympus, Japan). The percentage of lipid droplet area was determined using the ImageJ software.

### RNA extraction and qRT-PCR assay

BMMSCs at the second passage were collected, and total RNA was extracted by Trizol Reagent (Takara, Japan). RNA was reverse transcribed into cDNA by the reverse transcription kit (Takara, Japan). cDNA was amplified and signals were detected using the SYBR Green qPCR kit (Takara, Japan) on a BIO-RAD CFX96 real-time PCR system (Bio-Rad, USA) with sequence-specific primers shown below (5' to 3'). *Acsbg1*, Forward: ATGCCACGCGGTTCTGAAG; Reverse: GAGCTGGTTTGCGAGTTGTCT. *Nrap*, Forward: GTGATGAACCCAGAGGAAAGG; Reverse: GGGGTGTCTCACAGTAGTTGATA; *Rorc*, Forward: GACCCACACCTCACAAATTGA; Reverse: AGTAGGCCACATTACACTGCT. *Scart1*, Forward: CCTTGGCTGCTTTAGGAGAGG; Reverse: TAGGGCATAGAGACGGCTGT. *Shtn1*, Forward: CAGACCCAAGGCAAAGCCAG; Reverse: GTTCTCAGGGCCAAGGGATTT.* β-actin*, Forward: GGCTGTATTCCCCTCCATCG; Reverse: CCAGTTGGTAACAATGCCATGT. *β-actin* was used as a reference gene, and calculation was performed with the 2^-ΔΔCT^ method. Three independent qRT-PCR amplifications were performed for each sample. Expression values were quantified by the fold change over the Ctrl groups.

### Statistical analysis

Statistical analysis was performed to determine the significance of the data after assessing the normal distribution of data. The mean and standard deviation (SD) were calculated and presented. Statistical analysis is performed by two-tailed unpaired Student's t test for two-group comparison, with P < 0.05 indicating significance. Statistical analyses were conducted using the GraphPad Prism software (GraphPad Software, USA).

Note: all antibodies, chemicals, cytokines, plasmids, and software used in this study were listed in Table S1.

## Supplementary Material

Supplementary figure and table.

## Abbreviations

Acsbg1: Acyl-CoA synthetase bubblegum family member 1
AUC: area under the curve
BMD: bone mineral density
BMMSCs: bone marrow mesenchymal stem cells
BV/TV: bone volume over tissue volume
Ct.Ar: area of cortical bone
Ct.Th: thickness of cortical bone
CTX1: C-terminal cross-linked telopeptide 1
DEGs: differentially expressed genes
EDU: 5-ethynyl-2'-deoxyuridine
ELISA: enzyme-linked immunosorbent assay
FBS: fetal bovine serum
GEO: Gene Expression Omnibus
GO: Gene Ontology
GTT: glucose tolerance test
HbA1c: glycated hemoglobin
HFD: high-fat diet
IF: immunofluorescent
IFN-γ: interferon-gamma
ITT: insulin tolerance test
KEGG: Kyoto Encyclopedia of Genes and Genomes
Micro-CT: micro-computed tomography
NCD: normal chow diet
Nrap: Nebulin-related anchoring protein
ORO: oil red O
P1NP: procollagen type 1 N-terminal propeptide
PFA: paraformaldehyde
qRT-PCR: quantitative real-time polymerase chain reaction
ROI: region of interest
Rorc: RAR-related orphan receptor gamma
RUNX2: Runt-related transcription factor 2
Scart1: Scavenger receptor family member expressed on T cells 1
scRNA-seq: single-cell RNA-sequencing
SD: standard deviation
Shtn1: Shootin 1
T2D: type 2 diabetes
Tb.N: trabecular number
Tb.Sp: trabecular spacing
TNF-α: tumor necrosis factor-α
TRAP: Tartrate resistant acid phosphatase
UMAP: uniform manifold approximation and projection
