# Supplementary material for: Integrated Phenotypic and Transcriptomic Analyses of Osteoporosis in Type 2 Diabetic Mice
Source: Int J Med Sci. 2025 Mar 10;22(8):1773–90. doi: 10.7150/ijms.109537 (PMC11983309; doi:10.7150/ijms.109537)
Supplement: Supplementary file 1 — Supplementary figure and table. [file ijmsv22p1773s1.pdf]

Supplementary Figure and Legend

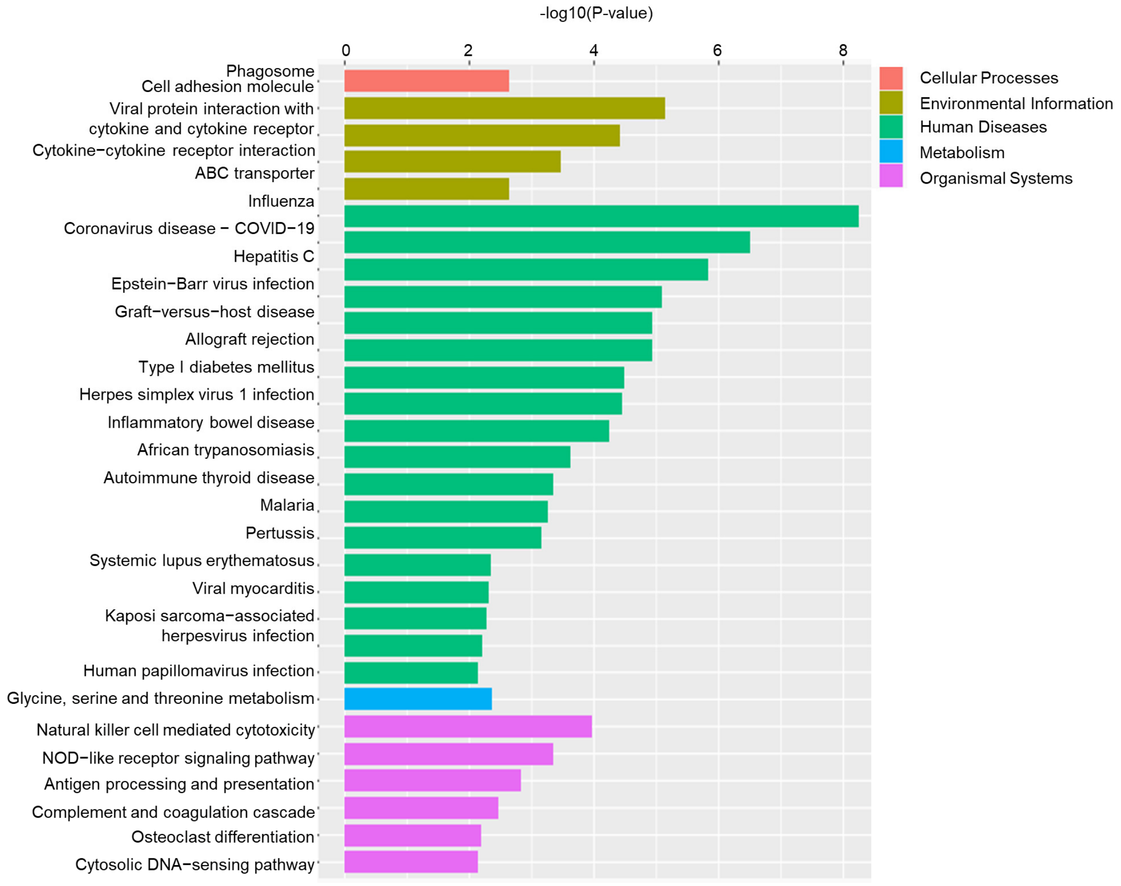

Figure S1. The top 30 pathways in KEGG enrichment analysis of differentially expressed genes (DEGs) in the whole bone marrow transcriptome at high-fat diet (HFD)-feeding for 4 weeks over control mice.

Table S1. Key resources table.

REAGENT or RESOURCE	SOURCE	IDENTIFIER
Antibodies		
RUNX2 (D1L7F) Rabbit mAb	Cell Signaling	Cat# 12556<br>RRID: AB_2800297
Anti-TRAP	Abcam	Cat# ab191406<br>RRID: AB_10555134
Anti-TNF-α (52B83)	Santa Cruz Biotechnology	Cat# sc-52746<br>RRID: AB_2924281
Donkey Anti-Rabbit IgG H&L (Alexa Fluor® 488)	Abcam	Cat# ab150073<br>RRID: AB_2636877
Cy3-AffiniPure Goat Anti-Rabbit IgG (H+L)	Yeasen	Cat# 33108ES60<br>RRID: AB_2338000
Alexa Flour 594 AffiniPure Goat Anti-Mouse IgG (H+L)	Yeasen	Cat# 33212ES60
Alexa Fluor® 488 anti-mouse F4/80	BioLegend	123120
Chemicals, peptides, and recombinant proteins		
Glucose	Sigma-Aldrich	Cat# G8270
Insulin	Novo Nordisk AS	N/A
4% paraformaldehyde	Biosharp	Cat# BL539A
Erythrocyte lysis buffer	Coollaber	Cat# SL1070
17% ethylene diamine tetraacetic acid (EDTA) decalcification solution	Proandy	Cat# 10218-1
Sucrose	Sigma-Aldrich	Cat# 57-50-1
Optimal cutting temperature (OCT) compound	Leica	Cat# 3801480
Triton X-100	Sigma-Aldrich	Cat# X100PC-5ml
Goat serum	BOSTER	Cat# AR0009
Mounting Medium With DAPI-Aqueous Fluoroshield	Abcam	Cat# ab104139
α-MEM	Invitrogen	Cat# 12571-048
Fetal bovine serum	Corning®	Cat# 35-081-CV
Penicillin-Streptomycin	Invitrogen	Cat# 15140-122
TrypLE™ express	Gibco	Cat# 12605028
Dexamethasone	Sigma-Aldrich	Cat# D4902
Ascorbic acid	MP Biomedicals	Cat# 100769
β-glycerophosphoric acid	Sigma-Aldrich	Cat# 154804-51-0
Penicillin-Streptomycin	Invitrogen	Cat# 15140-122
PBS	Invitrogen	Cat# 10010023
Indomethacin	MedChemExpress	Cat# HY-14397
Isobutyl methylxanthine	MedChemExpress	Cat# HY-12318
Crystal violet solution	Solarbio	Cat# G1063-100ml
Alizarin red S	Sigma-Aldrich	Cat# 130-22-3
Oil red O	Aladdin	Cat# O104972
kFluor488-EdU Cell Proliferation Test Kit (Imaging)	KeyGEN Biotech	Cat # KGA9602-100
Critical commercial assays		
RNeasy Mini Kit	Qiagen	Cat# 74106
NEBNext®Ultra™RNA Library Preparation Kit	New England Biolabs	Cat# E7770
HbA1c ELISA Kit	ENZO-2	NOV-BG-MUS11143-96T
Insulin ELISA Kit	Beyotime	P1602

Mouse CTX1 ELISA Kit	Fankew	F2808-A
Mouse P1NP ELISA Kit	Fankew	F2814-A
Trizol Reagent	Takara	9108
PrimeScript™RT reagent Kit	Takara	RR037A
TB Green® Premix Ex Taq™ II	Takara	RR820A
TruSeq PE Cluster Kit v3-cBot-HS	Illumia	Cat# PE-401-3001
Experimental models: Organisms		
Mouse: C57BL/6 mice	The Laboratory Animal Center of the Fourth Military Medical University	N/A
Software and algorithms		
Micview V2.1.2 software	GE Healthcare	N/A
Ilumina Hiseq platform	Ilumina	N/A
Hisat2 tool software	http://ccb.jhu.edu/software/hisat2/index.shtml	RRID: SCR_015530
DESeq	https://bioconductor.org/packages/release/bioc/html/DESeq2.html	RRID: SCR_015687
CellRanger software (version 3.1.0)	10x Genomics	RRID: SCR_023221
R language software	https://www.r-project.org/	RRID: SCR_001905
Seurat R package (version 3.0.1)	https://www.r-project.org/	RRID: SCR_001905
Pheatmap package (Version 1.0.12)	https://cran.r-project.org/web/packages/pheatmap/index.html	RRID: SCR_016418
ImageJ software	National Institute of Health	RRID: SCR_003070
GraphPad Prism software	GraphPad	RRID: SCR_002798
Other		
Rodent diet with 60% kcal fat	Research Diets Inc.	Cat# D12492
